# Evidence from Individual Inference for High-Dimensional Coexistence: Long-Term Experiments on Recruitment Response

**DOI:** 10.1371/journal.pone.0030050

**Published:** 2012-02-29

**Authors:** James S. Clark, Benjamin D. Soltoff, Amanda S. Powell, Quentin D. Read

**Affiliations:** 1 Nicholas School of the Environment, Duke University, Durham, North Carolina, United States of America; 2 Department of Biology, Duke University, Durham, North Carolina, United States of America; 3 Department of Statistical Science, Duke University, Durham, North Carolina, United States of America; Lakehead University, Canada

## Abstract

**Background:**

For competing species to coexist, individuals must compete more with others of the same species than with those of other species. Ecologists search for tradeoffs in how species might partition the environment. The negative correlations among competing species that would be indicative of tradeoffs are rarely observed. A recent analysis showed that evidence for partitioning the environment is available when responses are disaggregated to the individual scale, in terms of the covariance structure of responses to environmental variation. That study did not relate that variation to the variables to which individuals were responding. To understand how this pattern of variation is related to niche variables, we analyzed responses to canopy gaps, long viewed as a key variable responsible for species coexistence.

**Methodology/Principal Findings:**

A longitudinal intervention analysis of individual responses to experimental canopy gaps with 12 yr of pre-treatment and 8 yr post-treatment responses showed that species-level responses are positively correlated – species that grow fast on average in the understory also grow fast on average in response to gap formation. In other words, there is no tradeoff. However, the joint distribution of individual responses to understory and gap showed a negative correlation – species having individuals that respond most to gaps when previously growing slowly also have individuals that respond least to gaps when previously growing rapidly (e.g., *Morus rubra*), and vice versa (e.g., *Quercus prinus*).

**Conclusions/Significance:**

Because competition occurs at the individual scale, not the species scale, aggregated species-level parameters and correlations hide the species-level differences needed for coexistence. By disaggregating models to the scale at which the interaction occurs we show that individual variation provides insight for species differences.

## Introduction

Understanding how many competing species can coexist on few limiting resources remains one of the most important challenges for biodiversity science [1], [2], [3], [4], [5], [6]. Traditional niche theory might explain coexistence of only a few competitors on few limiting resources (e.g., light, water, several macronutrients), and it can do so only if i) there are strict tradeoffs in their responses to those resources and ii) the differences in these responses are large–there is ‘limiting similarity’. Niche differences could be most important at recruitment stages. If so, models indicate the need for strict tradeoffs in capacities to capture new sites early vs survive and grow in competition [7], [8], [9], [10]. Identifying species differences that promote survival in crowded, competitive environments or to find and occupy new sites in advance of competitors is viewed as critical to understanding the rich diversity of forest communities [11], [12], [13], [14], [15]. Although the evidence for high diversity of competitors is ubiquitous [2], evidence for the strict tradeoffs needed to predict that diversity in models is not. Where such tradeoffs are evident they surely contribute to coexistence, but they do not emerge for many of the species examined in field studies [5], [16].

Clark [16] noted that the species differences required for coexistence need not be apparent in the species-level aggregate parameter values that are estimated in empirical studies and implemented in theoretical models. Using disaggregated (individual-level) data he showed that dynamics are consistent with high-dimensional coexistence. Individuals respond to spatio-temporal variation more like others of the same species, thus concentrating competition within the species. The disaggregation to the individual scale is motivated by the fact that individuals compete, whereas species do not. Differences between species that are missed in traditional analyses can be quantified using individual-scale inference that considers a joint distribution of responses to environmental variables [17]. But studies have not yet shown how the disaggregated (individuals within species) relationships differ from the species aggregates. If disaggregation is critical to understanding coexistence in a high-dimensional environment, it is important to understand how individual level data change the interpretation of species differences. Using a 20-yr experiment to determine how individuals of different species respond to recruitment opportunities following canopy gap formation we show that disaggregation transforms the interpretation from one that would not promote coexistence in aggregate, but one that does at the individual scale.

To illustrate how individual-level data can change coexistence criteria Clark et al. [18] showed some of the many ways interpretations change when moving from the aggregate species-level parameters to a joint distribution of individuals. The issues involved are long recognized in a well-developed literature in statistics and the social sciences termed the ‘ecological fallacy’ [19], [20]. Because recruitment, competition, and risk behavior operate at the scale of individuals, aggregated responses hide or even change important relationships. The problem comes from the fact that the joint distribution is lost in the marginalization over individuals to obtain the species aggregate.

**Figure 1:**
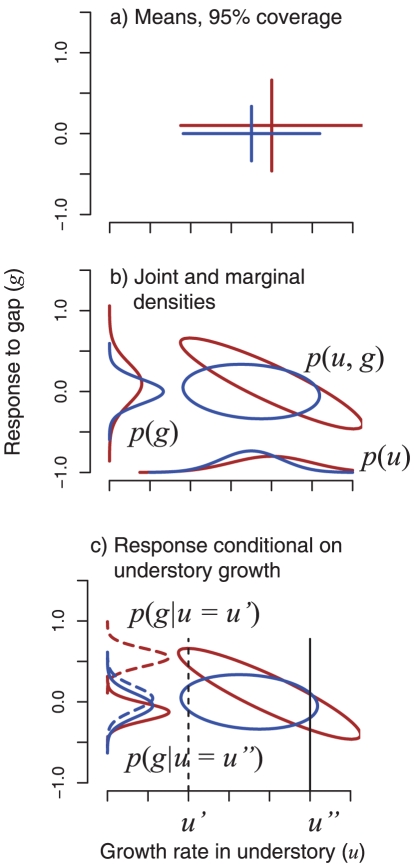
Effect of aggregation on species inference. a) Crosshairs show means and 2 standard deviations for two species plotted on two niche axes, with brown dominating blue. The examples are understory (*u*) vs. gap (*g*) response. b) Joint distributions of individuals in response to the same variables are the basis for crosshairs in part (a). The aggregate summaries by species are shown as marginal distributions along the margins. The brown species dominates both. c) Conditional distributions show the brown dominating the gap response at low understory growth (*u′*) and blue dominating at high understory growth (*u″*).

Figure 1 shows an example of two hypothetical species responding to two different environmental settings, understory *u* and gap *g*. The standard comparison for two traits, aggregate means and 95% marginal coverage, are shown in Figure 1a. The positive correlation between the two species is not consistent with tradeoffs that would promote coexistence–the brown species dominates in both environments. The problem with this interpretation is that individuals, not species, compete. The aggregate summaries miss the structure, contained in joint distributions of individuals, shown in Figure 1b. Because individuals compete, the conditional distribution is the relevant scale for the interaction. At the individual scale, the stronger gap response belongs to the brown species only when or where understory growth is slow. The blue species has the stronger response when understory growth is rapid (Fig. 1c). Whereas the aggregate summaries would appear to exclude understory vs gap response as an important difference for maintaining coexistence (Fig. 1a), the joint distribution of individuals shows that this interpretation misses the combinations of responses where either species could win, depending on understory growth rates of each. This is an example of Simpson's Paradox [21], [22], essentially ignored in ecology, but recognized as one of the central considerations when interpreting social sciences and public health data [23], [24]. Clark et al [18] demonstrate with a large number of examples why the aggregation problem is widespread in ecology. They discuss why disaggregation by individual and year provides evidence for its importance in coexistence studies, in terms of correlation structure. The question now is, do the joint distributions of responses differ among species and, if so, can those differences provide insight about coexistence?

In this paper we test for individual-scale responses of twenty species to the recruitment opportunities represented by canopy gap formation. The approach follows a long tradition that attempts to identify tradeoffs that operate in a small number of dimensions, in this case understory vs gap growth response [11], [12], [15], [25], [26], [27]. Our goal is not to determine if this is *the* tradeoff that regulates diversity in a specific forest–we have shown that species differ in their responses to many variables in these stands [17], [28], and each provides opportunities for species to partition the environment. While it is possible to identify patterns that should contribute to coexistence, it is not possible to indentify all mechanisms that contribute and exclude all of those that do not. We are not attempting define what causes coexistence. We address the long-standing challenge to identify what could contribute to coexistence. Specifically we address the general question of how a joint distribution of individuals changes the interpretation of such studies. An intervention design allowed us to observe pre- and post-treatment responses of individuals exposed to canopy gaps, followed for eight post-treatment years. We examine the relationship between understory growth rate and change in growth rate following gap formation, the latter because we found that the post-gap response was best described by a trend rather than a fixed value. Each year following gap formation there is a different growth rate. Of course, we do not expect a trend to persist indefinitely, but it best describes the response at this scale.

A hierarchical Bayes model yields inference on the joint distribution of individuals within species, allowing us to compare responses inferred at both levels. We show that the positive correlation between species aggregate responses to understory and gap formation reverses at the disaggregated scale, with species having individuals that respond most to gaps when previously growing slowly in the understory are often those having individuals that respond least when previously growing rapidly before gap formation. The disaggregated result lends support to a role for canopy gaps contributing to coexistence, whereas the aggregated result does not.

The Methods section of this paper describes how data on responses to understory and gap conditions were obtained at the individual scale and used to infer joint distributions of responses, like those in Figure 1a. The posterior distribution of responses from our hierarchical model is disaggregated in a conditional sense to examine the relevant scale of interactions as in Figure 1c. We begin by summarizing why the joint distribution of individuals is critical for understanding biodiversity regulation.

### A joint distribution of individuals broadens potential for species coexistence

As with any analysis of tradeoffs, ours cannot demonstrate that a particular parameter relationship (e.g., a negative correlation among species in terms of their mean understory growth and gap response) is responsible for coexistence. The important literature on this topic has instead shown that there is sometimes evidence for tradeoffs of the type that would be necessary for coexistence. In competition models, tradeoffs are necessary, but not sufficient for coexistence. They are necessary for coexistence in models that include only a few niche variables (e.g., resources, regeneration sites, or colonization-competition tradeoffs), because only with tradeoffs can many species partition a low dimensional niche space, such that each finds opportunity to succeed [3], [7], [8]. A tradeoff, interpreted from a negative correlation among species in terms of success under different conditions, is not a sufficient explanation, because there is requirement for limiting similarity [8], [9], [16]. Simply stated, this means that there is a delicate balance required for many species to coexist in a low dimensional environment, a balance that is hard to obtain in models. Correlations reported in the literature typically would not be sufficient to explain coexistence, because they constitute a rough trend, but would not satisfy a limiting similarity requirement. Demonstration that an observed trend controls diversity would be infeasible outside multiple generation studies under tightly controlled experimental conditions, conditions that might have little relevance to field settings. Still, such trends in observational data probably contribute to diversity, despite being insufficient to explain coexistence on their own.

The delicate balance (strict parameter relationship) required for coexistence in models is an artifact of the assumption that only a few dimensions affect species interactions [5]. If there are many ways to succeed that differ among species, then coexistence is not hard to explain. Coexistence of one gap and one understory specialist is easy in an environment limited to ‘gap’ vs ‘understory’. A delicate balance is required if we attempt to explain coexistence of many species in this simple environment. The need for a delicate balance is removed if there are more dimensions to partition. There is no problem explaining coexistence of many species, if there are many ways to partition the environment [1], [5].

The environment is high dimensional [1], but the potential importance is hidden and even misrepresented in species aggregate parameters [16], [18]. Only light, moisture, and a few macronutrients emerge as generally limiting in studies of plant diversity at the aggregate species level, thus motivating the search for a low-dimensional explanation of diversity [reviewed by 27]. However, the effects of these few variables are supplemented and modulated by a large number of other variables and their interactions. The importance of these effects emerges from the joint distribution of individuals [16]. The apparent winner in Figure 1b (i.e., having the largest mean response) would apply to the case where species, rather than individuals, interact. In Figure 1a, the individual winners for *x* change with *y*, and vice versa. If *y* is unobserved, and only the species aggregate distribution is available, there would be no way to explain why sometimes blue wins and sometimes brown wins, i.e., the basic requirement for coexistence. In this study we do not demonstrate that individuals ‘explain coexistence’. Rather we provide the more important evidence that the individual perspective removes the delicate balance necessary for coexistence in models where the assumption is that species, rather than individuals, compete. We show that under a simple dichotomy of ‘gap’ vs ‘understory’, the joint distribution of individuals provides multiple ways for different species to win, whereas the species aggregate values do not.

## Results

**Figure 2:**
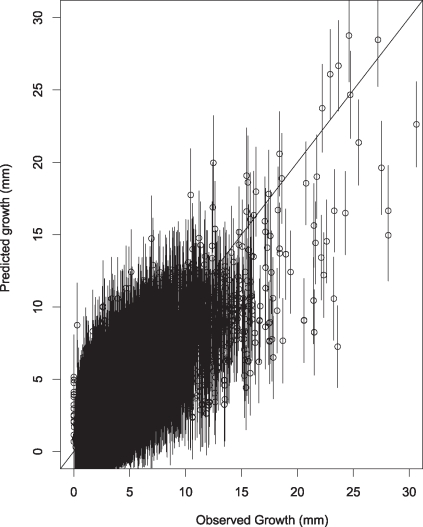
Predictive check on fitted model. Model predictions for 25,787 growth rates of all species are accurate even for the rare high values, despite the fact that observations are primarily in the low range.

We obtained good predictive capacity across the full range of growth rates in our study using a model that included a random effects covariance matrix on understory and gap responses (Fig. 2). With only a random effect on gap responses (not shown) the model did not accurately predict the lowest and highest growth rates. In other words there are relationships among individuals within each species in terms of how they respond to the two environments, described by a mean vector of responses and covariance for the species (eqn 2).

**Figure 3:**
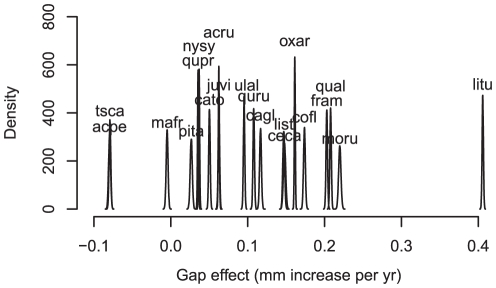
Posterior estimates of gap response. Gap response in terms of the per-yr enhancement of growth over understory individuals of the same species. These are posterior densities for parameters *α_s,g_*.

Credible intervals for species-level growth responses to gaps (see Table S1) overlap for only two pairs of species (*Nyssa sylvatica*:*Quercus prinus* and *Tsuga canadensis*:*Acer pennsylvatica*), and show a clear ranking from the fast-responding *Liriodendron tulipifera* to negative responses for *Tsuga* and *A. pennsylvatica* (Fig. 3). The change in growth rate per year is plotted in Figure 3, because there was a trend in the response. In the case of *Tsuga*, the post-gap period corresponds to the hemlock adelgid expansion into our region, thus explaining apparent negative response. The highly shade-tolerant *A. pensylvanicum* not only had the lowest gap response, but also had the slowest average growth rate (Fig. 3).

**Figure 4:**
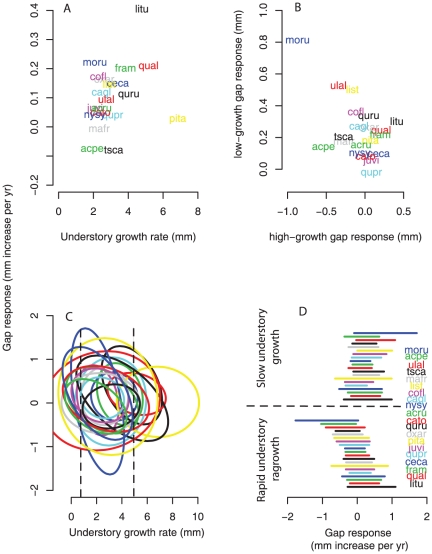
Joint distribution of gap and understory response. Species level means, showing positive correlation in aggregate for *α_s,u_* and *α_s,g_* (a), negative correlation in the conditional means for low understory growth rate 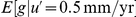 and high understory growth 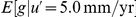 (b), and joint distributions of individuals within species *p*(*g, u*) (c). The joint distributions in (c) are from eqn 2, where vertical dashed lines indicate the conditional understory growth rates used to plot (b). The 95% conditional intervals are shown in (d).

At the species level, the correlation between responses to understory and gap is positive (*r* = 0.29, *P* = 0.22) and strongly positive if we exclude species *Pinus taeda* (*r* = 0.65, *P* = 0.003)(Fig. 4a), which did not actually have individuals in the understory prior to gap formation. In other words, all *P. taeda* trees were exposed to direct sunlight, regardless of treatment. Thus, the positive relationship is strong and not consistent with negative correlation needed to promote coexistence.

Disaggregation changes the interpretation, showing a negative relationship between gap responses for individuals previously growing slowly or rapidly in the understory (*r* = −0.51, *P* = 0.023) (Fig. 4b). Species having the individuals that respond most to gaps when growing rapidly in the understory are not the same species having individuals that respond most when growing slowly in the understory. The joint distributions of individual responses (eqn 2) are shown as 95% ellipses in Figure 4c. The 95% intervals (Fig. 4d), with species presented in the same order for both conditional responses, show the reverse tendencies for the two groups. This pattern can arise if there is a maximum growth rate for the species, so that the gap response has to be lower for individuals already growing near that rate. However, this effect is not sufficient to explain our results, because the range of variation in gap responses is as large as the range of understory responses (Fig. 4c).

## Discussion

The species aggregate relationships from field evidence are not consistent with model predictions that would be necessary for coexistence of large numbers of competitors on a few limited resources. Where species-level tradeoffs exist, they almost surely contribute to coexistence, but they are frequently lacking and rarely could they meet the requirement of limiting similarity [16]. Tilman [8] shows evidence for a species-level tradeoff consistent with coexistence of five competitors. Adler et al. [6] provide simulation results for a fitted model suggesting coexistence of four competitors. Angert et al. [29] show that tradeoffs between water use efficiency and growth likely contribute to coexistence of 11 desert annual species. The challenge of explaining coexistence of dozens to thousands of competitors in crowded canopies remains daunting. In this study there is a clear ranking of species in terms of gap response, when viewed as the standard species parameter estimates (Fig. 3) and positive correlation with performance in the understory (Fig. 4a). Although previous work agrees with the species-level aggregate responses we report, they do not provide a basis for comparison of individual level responses, i.e., the relevant scale for competition. *Liriodendron tulipifera* tend to colonize large gaps [11], [30], [31] where they have high growth rates [13], [16]. In greenhouse experiments, *Liriodendron* seedlings respond to high light and high-nutrient conditions, whereas *Carya tomentosa*, *Nyssa sylvatica*, and *Quercus rubra* show more modest differences [32]. Large gaps like those in our study favor shade-intolerant species [33], [34], [35], [36], but with exceptions [5], [37], and gaps smaller than those examined here could favor *A. pennsylvanicum* [38]. If the same species do best in both low and high light, then gap formation, at least when viewed in aggregate, does not provide evidence for coexistence [5], [26], [11]. However, disaggregation shows this marginal view to be a distortion, brought on by aggregating over the scale at which competition for gaps occurs, i.e., individuals competing for light in the context of other variables. Disaggregation changes inference by showing that situations favoring understory and gap success at the individual level differ by species.

Given that tradeoffs are sometimes observed at the species level, but often not, raises the question of when the individual scale will differ from the species scale. The marginal (species) distribution is the same as the conditional (individual) distribution when there are no unmeasured variables that affect the response. When this is the case, no additional detail emerges from the conditional view. Given the many and profound physiological and functional differences in responses to environmental variables, large differences between conditional and marginal responses are to be expected.

The conditional distributions (Fig. 4c, d) are the relevant perspective for evaluating species relationships, coming from the scale where the process operates–individuals responding to their local canopy environment. The aggregate species scale suggests that the same species dominate understory and gaps. The joint distribution of individuals reveals that the species having individuals that respond most to gaps when growing slowly in the understory are not the same as those that respond most to gaps when growing more rapidly in the understory–the fastest growing individuals in the understory do not necessarily show the largest gap responses. Despite the fact that *Morus* and *Ulmus* individuals grow more slowly on average than *Liriodendron* in both settings, they respond more strongly to gaps when previously growing slowly in the understory than did *Liriodendron* (Fig. 4c). In other words, situations where *Morus* and *Ulmus* perform poorly in the understory allow them to exploit gaps (Fig. 4c, d). The negative relationship in Figure 4b does not in itself explain coexistence, but demonstrates that there are ways to partition gaps that cannot be inferred from the aggregate mean values of Figure 4a.

Our goal in this analysis was not to identify the specific variables and interactions that explain persistence of a given species, or coexistence of them all. That would not be possible, nor would it have generality for other species in other forests. Rather, we demonstrate the more general point, how the joint distribution of individuals has relevance for the widespread efforts to identify the low dimensional tradeoffs viewed as critical for explaining forest diversity. The joint distributions in Figure 4c underlie the tendency for individuals to respond to the environment more like others of the same species, and they result from differences that can be measured [28]. The problem is that environmental information is typically limited to a few variables or none at all (most tree demographic studies compare average rates, rather than responses to environmental variables). If we fit the model to species level parameters, there is no evidence for environmental partitioning (Fig. 4a). By contrast, the joint distribution of responses for a species provides evidence of hidden dimensionality. Understory growth rate and gap response are influenced by soil moisture, light, winter temperature, summer drought, and spatial climate variation. All of these variables and their interactions have large effects on individual growth of species in this study [17], [28], and they contribute to joint distributions in Figure 4c–they influence how individuals of a species respond to canopy gaps.

Consider first how a single hidden variable can contribute to this joint distribution (we use soil moisture initially), followed by a large number of them [18]. Understory growth rates can be especially low for light-demanding species on moist sites where leaf area is high and, thus, there is deep shade. Despite being limited by moisture, seedlings of many species are more abundant on dry rather than wet sites due to the higher light availability. Responses to gap creation can be especially large on moist sites due to the combination of high light and moisture, which becomes available with loss of nearby mature trees. This combination can explain a negative correlation between understory growth and gap response within a species, due to the hidden variable, soil moisture.

Now consider how this joint perspective influences the interpretation of species differences. A more shade tolerant species grows more rapidly on moist sites (it can exploit low light and high moisture) and responds less to gap creation. In contrast to a light-demanding species, its slow understory growth rates are associated with dry rather than wet sites. Gap creation on dry sites produces a smaller increase in light and soil moisture than occurs on moist sites [11], and there is less response. The combination of a large negative correlation for the light-demanding species and a weak or zero correlation for the shade tolerant species means that the species responding most to gaps reverse from low to high understory growth rates.

The differences in response revealed by the joint distribution of individuals contribute to coexistence by concentrating competition within the species [16], and measured variables show why [28]. This becomes apparent when expanding the perspective beyond light and moisture. *Pinus taeda* and *Liquidambar* have similar mean growth rates and both benefit from high light. However, individuals of *P. taeda* exploit warm winters, whereas *Liquidambar* do not, and *P. taeda* suffers more from summer drought than *Liquidambar*. These ‘main effects’ and their interactions provide a high-dimensional set of constraints that can be partitioned [18] in ways are lost and even misrepresented in species-level comparisons in one or two dimensions [28]. This high-dimensional set of constraints explains the fact that individual responses are correlated most with others of the same species [16] and why the explanations based on a few limiting resources do not.

The fact that responses to light and moisture can be modulated by many variables will not be news to field ecologists. Physiological ecologists have long recognized that responses to the environment are complex. Population and community ecologists have not recognized that this complexity is the explanation for diversity, arguing instead that patterns as widespread as succession requires a general explanation, and generality must be simple. The problem has been to understand why model predictions for coexistence are not consonant with results from field studies. Biodiversity is not explained by a few variables (e.g., light, soil moisture), despite the fact that these are the only variables that emerge as ‘limiting’ when viewed from the aggregate species perspective. This analysis demonstrates the change in perspective provided by the joint distribution, even without benefit from actually observing other niche axes. The reversal in success from species-level (Fig. 4a) to individual-level (Fig 4b) or alternatively from low to high understory growth rate (Fig. 4d) does not demonstrate that this is the mechanism for coexistence. No pattern in traits like this could identify a single mechanism for coexistence, because we only observe a few dimensions, and all can contribute. It does demonstrate a more general and important relationship. The joint distributions of Figure 4c result from variation within the two environments, but translated differently by different species. Neither gaps nor understory are homogeneous, supporting a range of light, drainage, and parent material [17]. The joint distribution of responses to that variation differs among species [18]. The role of unmeasured variables is consistent with previous studies showing the importance of interactions among variables [28], [39], [40], [41]. Our approach demonstrates that species differences that could contribute to coexistence are recognizable even when information on factors responsible for those differences are lacking. The standard practice of summarizing relationships with marginal means and standard deviations (Fig. 1b, 4a) hides and distorts relationships that are recovered by disaggregation.

## Methods

The analysis consists of an intervention design, where trees of similar size and canopy architecture were assigned to control/treatment pairs, one of the pair subjected to canopy opening. A hierarchical Bayes analysis was implemented to infer the joint and marginal distributions of responses to gap and understory as in the Figure 1 example.

### Design

The gap experimental methods are detailed in Dietze and Clark [42]. Mapped stands of mature forest at Coweeta Hydrologic Laboratory in the southern Appalachians (35°03′N, 83°27′W) and the Duke Forest in the North Carolina Piedmont (35°85′N, 79°05′W) were established in 1999. Field sites are owned by the US Forest Service and Duke University. Permissions for field sampling were obtained by James S. Clark and granted by Judd Edeburn (Duke University) and Jim Vose (USFS). Field studies did not involve endangered or protected species.

Individuals of 20 dominant species were identified as gap treatment-control pairs matched by diameter and canopy exposure levels. Pretreatment sampling began in 1999 (next section). Experimental gaps were created in March 2002 by pulling trees with a skidder, a technique reported by Cooper-Ellis et al. [43]. Trees were left in place, consistent with wind damage in these stands. A total of eight 20 m and ten 40 m diameter gaps were created at the two sites. Gap treatment trees occupy the edges of the gap following treatment and thus are broadly exposed to full sunlight. They range up to 20 cm in diameter. Details on these sites are provided in [5]. The number of trees and tree years in this study are shown in Table 1.

**Table 1:** Numbers of trees and tree years in the study by species.

*Species*	Understory trees	Gap trees	Understory years	Gap years
Acer pensylvanicum (acpe)	9	13	332	104
Acer rubrum (acru)	114	268	5563	2133
Carya glabra (cagl)	23	22	769	176
Carya tomentosa (cato)	25	43	1038	344
Cercis canadensis (ceca)	5	11	185	78
Cornus florida (cofl)	11	23	384	180
Fraxinus americana (fram)	10	19	431	148
Juniperus virginiana (juvi)	13	29	616	232
Liquidambar styraciflua (list)	11	14	397	112
Liriodendron tulipifera (litu)	39	68	1601	537
Magnolia fraseri (mafr)	11	19	424	152
Morus rubra (moru)	8	7	234	53
Nyssa sylvatica (nysy)	22	65	1285	520
Oxydendrum arboretum (oxar)	7	37	580	296
Pinus taeda (pita)	6	9	243	72
Quercus alba (qual)	19	32	815	256
Quercus prinus (qupr)	22	63	1281	504
Quercus rubra (quru)	23	35	938	280
Tsuga canadensis (tsca)	33	37	1134	296
Ulmus alata (ulal)	21	41	755	309

### Growth rate methods

Trees were measured for diameter at breast height at 2 yr intervals. For detailed analysis of gap effects we selected individuals at the gap edge and from the forest interior to produce a balanced design, with similar sample sizes of gap and non-gap trees (Table 1).

Increment core samples and diameter measurements were collected in 2010 from all trees greater than 5 cm in diameter within and at the edges of canopy gaps. Resprouts from downed canopy trees within the gaps were not included. Each gap tree was paired with an individual of the same size and species close to the gap tree, but unaffected by gap creation. Non-gap individuals were sampled in the same manner as gap trees. From one to three increment cores were obtained from each tree. Cores were mounted onto wooden blanks, sanded, and analyzed using a stage micrometer, producing a record of annual growth as basis for subsequent analysis.

### Inference

Model objectives included inference on the joint distribution of responses in gaps *g* and understory *u*. We analyzed growth beginning from 1990. Each individual began the experiment in the understory. Half became exposed to canopy gaps in 2002. We estimated effects on tree growth using a random effects model that includes both a random understory and gap response,
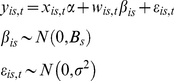
(1)where the design vector *x_is,t_* includes indicators for the species-*s* understory growth rate, with corresponding parameter *α_s,u_*, whether or not individual *i* is a gap tree, with parameter *α*_0_, and the number of years *t* since the gap was created, with parameter *α_s,g_*. The length-41 parameter vector is
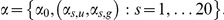
The response *y_is,t_* is growth rate of tree *i* of species *s* in year *t* in cm per year. The indicator for whether a tree received the gap treatment is constant for the entire study interval and takes up differences between individuals in the treatment groups not accounted for by the gap-control treatment itself [44]. The time since gap creation is zero for trees not receiving the gap treatment. For trees receiving the gap treatment there are zeros from 1990 to 2002, followed by an increment of 1 year annually, i.e., from 1 to 8. The corresponding ‘gap response’ parameter *α_s,g_* thus represents the annual rate of increase in growth rate over understory rates. This design, rather than a step function (i.e., all post gap years receive a 1), was used because it best described the data. The random effects vector *w_is,t_* include all inputs contained in *x_is,t_* except whether or not the individual is a gap tree. Gap responses can be negative, as when individuals suffer from sudden exposure to high light and increased leaf temperatures, but are predominantly positive. Eqn 1 represents a hierarchical model, discussed in detail in [5], [45].

Of particular interest in this analysis is the individual variation within species, which allows us to evaluate relationships summarized in Figure 1. The random effects covariance matrix *B_s_* differs for each species *s*, describing the joint distribution of understory *u* and gap response *g*

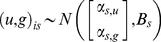
(2)where *B_s_* is the covariance matrix having variances *B_s(u)_* and *B_s(g)_* on the diagonal and covariance *B_s(u,g)_*. Random treatment of both responses provided good predictive capacity of low and high growth rates (see Results), capturing the fact that there is large variation in how individuals of a species grow in both environments. To understand how this joint distribution influences the individual level relationships we further examine conditional relationships for growth responses at different understory growth rates *u′*,
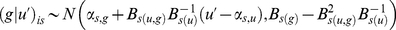
(3)The conditional distribution allows us to determine differences in gap responses for individuals growing at different rates in the understory across species. This is the relevant scale for evaluating species differences (Fig. 1c).

Prior distributions and parameter values are non-informative
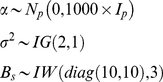
(4)Posterior simulation was accomplished with Gibbs sampling written in R, using methods detailed in [5], [44].

## Supporting Information

Table S1 **Posterior percentiles for parameters**: This table contains posterior means and marginal 95% credible intervals for parameter values.
(DOCX)
