# Supplementary material for: Evidence from Individual Inference for High-Dimensional Coexistence: Long-Term Experiments on Recruitment Response
Source: PLoS One. 2012 Feb 29;7(2):e30050. doi: 10.1371/journal.pone.0030050 (PMC3290613; doi:10.1371/journal.pone.0030050)
Supplement: Table S1 — Posterior percentiles for parameters: This table contains posterior means and marginal 95% credible intervals for parameter values. (DOCX) [file pone.0030050.s001.docx]

Posterior percentiles for parameters

|  | Posterior 50% 2.5% 97.5% |
| --- | --- |
| α_0_ | Gap -0.0378 -0.0577 -0.0176 |
| α_s,u_ | acpe 1.93 1.92 1.94 |
|  | acru 2.45 2.44 2.46 |
|  | cagl 2.45 2.45 2.46 |
|  | cato 2.41 2.4 2.42 |
|  | ceca 3.40 3.39 3.41 |
|  | cofl 2.27 2.26 2.28 |
|  | fram 3.84 3.83 3.85 |
|  | juvi 2.09 2.08 2.11 |
|  | list 2.9 2.89 2.91 |
|  | litu 4.78 4.77 4.79 |
|  | mafr 2.32 2.31 2.33 |
|  | moru 2.08 2.07 2.09 |
|  | nysy 2.08 2.06 2.09 |
|  | oxar 2.62 2.61 2.64 |
|  | pita 6.84 6.83 6.85 |
|  | qual 5.17 5.16 5.18 |
|  | qupr 3.1 3.09 3.11 |
|  | quru 4.03 4.02 4.04 |
|  | tsca 3.16 3.15 3.17 |
|  | ulal 2.74 2.73 2.75 |
| α_s,g_ | acpe -0.0796 -0.0822 -0.0771 |
|  | acru 0.0625 0.0611 0.0639 |
|  | cagl 0.117 0.114 0.119 |
|  | cato 0.0503 0.0484 0.0521 |
|  | ceca 0.148 0.145 0.151 |
|  | cofl 0.174 0.171 0.176 |
|  | fram 0.203 0.201 0.205 |
|  | juvi 0.0626 0.0607 0.0645 |
|  | list 0.147 0.145 0.149 |
|  | litu 0.406 0.404 0.407 |
|  | mafr -0.00481 -0.00724 -0.0024 |
|  | moru 0.22 0.217 0.223 |
|  | nysy 0.0368 0.0354 0.0382 |
|  | oxar 0.161 0.16 0.163 |
|  | pita 0.0266 0.0239 0.0293 |
|  | qual 0.208 0.206 0.21 |
|  | qupr 0.0355 0.0341 0.0368 |
|  | quru 0.108 0.106 0.11 |
|  | tsca -0.0792 -0.0813 -0.077 |
|  | ulal 0.0953 0.0935 0.0971 |
| *B_s(u)_* | acpe 1.91 1.08 3.74 |
|  | acru 1.95 1.68 2.26 |
|  | cagl 1.16 0.779 1.91 |
|  | cato 1.24 0.859 1.78 |
|  | ceca 2.55 1.22 6.47 |
|  | cofl 0.981 0.555 1.85 |
|  | fram 3.75 2.14 6.49 |
|  | juvi 1.26 0.776 2.03 |
|  | list 4.56 2.69 8.06 |
|  | litu 7.79 5.96 10.7 |
|  | mafr 2.73 1.63 4.99 |
|  | moru 1.48 0.669 4.47 |
|  | nysy 1.06 0.777 1.54 |
|  | oxar 1.84 1.27 2.87 |
|  | pita 5.10 2.60 11.0 |
|  | qual 3.44 2.37 5.26 |
|  | qupr 1.90 1.39 2.62 |
|  | quru 4.23 2.91 6.05 |
|  | tsca 2.17 1.58 3.16 |
|  | ulal 2.76 1.92 4.12 |
| B_s(u,g)_ | acpe -0.258 -0.732 0.0221 |
|  | acru -0.0907 -0.153 -0.036 |
|  | cagl -0.0922 -0.293 0.114 |
|  | cato -0.0257 -0.158 0.0982 |
|  | ceca 0.0497 -0.487 0.65 |
|  | cofl -0.103 -0.428 0.0867 |
|  | fram -0.0587 -0.618 0.351 |
|  | juvi 0.00942 -0.158 0.174 |
|  | list -0.671 -1.42 -0.201 |
|  | litu 0.155 -0.339 0.653 |
|  | mafr -0.283 -0.673 0.012 |
|  | moru -0.505 -2.02 0.126 |
|  | nysy -0.0364 -0.118 0.0365 |
|  | oxar -0.08 -0.296 0.0923 |
|  | pita -0.24 -1.57 0.925 |
|  | qual -0.0281 -0.328 0.338 |
|  | qupr 0.0446 -0.0444 0.148 |
|  | quru -0.247 -0.614 0.0159 |
|  | tsca -0.23 -0.459 -0.0693 |
|  | ulal -0.513 -0.977 -0.194 |
| *B_s(g)_* | acpe 0.266 0.148 0.541 |
|  | acru 0.0845 0.0700 0.105 |
|  | cagl 0.185 0.107 0.325 |
|  | cato 0.107 0.0684 0.172 |
|  | ceca 0.336 0.169 0.864 |
|  | cofl 0.226 0.139 0.444 |
|  | fram 0.23 0.128 0.439 |
|  | juvi 0.155 0.0967 0.279 |
|  | list 0.322 0.183 0.68 |
|  | litu 0.474 0.355 0.657 |
|  | mafr 0.21 0.125 0.426 |
|  | moru 0.884 0.402 2.28 |
|  | nysy 0.0776 0.0551 0.113 |
|  | oxar 0.141 0.0924 0.222 |
|  | pita 0.542 0.276 1.47 |
|  | qual 0.171 0.107 0.29 |
|  | qupr 0.091 0.0663 0.131 |
|  | quru 0.203 0.125 0.321 |
|  | tsca 0.134 0.0895 0.221 |
|  | ulal 0.400 0.258 0.633 |
| *σ*^2^ | 2.03 2.00 2.07 |
